# Decidual RANKL/RANK interaction promotes the residence and polarization of TGF-β1-producing regulatory γδ T cells

**DOI:** 10.1038/s41419-019-1380-0

**Published:** 2019-02-08

**Authors:** Rui-Qi Chang, Jun Shao, Yu-Han Meng, Jian Wang, Da-Jin Li, Ming-Qing Li

**Affiliations:** 1 Key Laboratory of Reproduction Regulation of NPFPC, SIPPR, IRD, Hospital of Obstetrics and Gynecology, Fudan University Shanghai Medical College, 200011 Shanghai, People’s Republic of China; 2 Department of Gynecology, Hospital of Obstetrics and Gynecology, Fudan University, 200011 Shanghai, People’s Republic of China; 3 Reproductive Medical Center, Affiliated Hospital of Weifang Medical University, 261030 Weifang, People’s Republic of China; 4 Shanghai Key Laboratory of Female Reproductive Endocrine Related Diseases, Hospital of Obstetrics and Gynecology, Fudan University Shanghai Medical College, 200011 Shanghai, People’s Republic of China

## Abstract

Decidual γδΤ (dγδΤ) cells play an essential role during successful pregnancy; however, the residence and polarization of γδΤ cells in decidua remain unclear. In this study, we observed higher levels of receptor activator for nuclear factor-κ B ligand (RANKL) on decidual stromal cells (DSCs), and its receptor RANK on dγδΤ cells in decidua from normal pregnancy compared with patients with recurrent spontaneous abortion (RSA). RANKL expressed by DSCs can induce the polarization of peripheral blood γδΤ (pγδΤ) and dγδΤ cells to Foxp3 + γδΤ cells, and upregulate the expression of transforming growth factor (TGF)-β1. This process is mediated through activation of nuclear factor kappa-light-chain-enhancer of activated B cells (NF-κB). In addition, RANKL promotes the adhesion of dγδΤ cells to DSCs in vitro, which is associated with the upregulation of ICAM-1 and VCAM-1 on DSCs and integrins on dγδΤ cells. RANKL knockout leads to the decreased numbers of uterus total γδΤ cells, Foxp3+γδΤ cells and the expression of TGF-β1, and the increased pregnancy loss in mice. These results suggest that RANKL is a pivotal regulator of maternal-fetal tolerance by triggering the polarization and residence of TGF-β1-producing Foxp3+γδΤ cells in early pregnancy. The abnormal low level of RANKL/RANK results in pregnancy loss because of the dialogue disorder between DSCs and dγδΤ cells. This observation provides a scientific basis on which a potential marker can be detected to early warning of pregnancy loss.

## Introduction

Decidual immune cell (DIC), one of the major components at the maternal-fetal interface, is critical in the induction of maternal immune tolerance to fetal alloantigen during pregnancy^1–3^. Abnormity of DIC is related to several pathological pregnancies, including recurrent spontaneous abortion (RSA), unexplained infertility, preeclampsia, and intrauterine growth restriction (IUGR)^4,5^. Decidual γδ T (dγδ T) cells, accounted for over 60% of T cells in human decidua, participate in maintenance of pregnancy by recognizing alloantigen without MHC restriction, producing cytokines and linking the innate and adaptive immune responses as a bridge^6–8^.

Similar to CD4 helper T (Th) cells, γδ T cells can be polarized toward six distinct subgroups upon activation based on their functional and developmental features^9,10^. γδ1, 2, 17, 22, follicular helper (FH), and regulatory (reg) cells are characterized by its capacity to produce interferon (IFN)-γ, interleukin (IL)-4, IL-17, IL-22, Th2-cell-associated cytokines (including IL-4 and IL-10), and transforming growth factor (TGF)-β, respectively. Moreover, T-bet, GATA‐binding protein 3 (GATA3), RORC, Bcl-6, and Foxp3 are the master transcription factors for the polarization of γδ1, 2, 17, FH, and reg, respectively^11–15^. Accumulating evidence showed that dγδ T cells have a tendency to secrete immunosuppressive cytokines, especially TGF-β and IL-10 at maternal-fetal interface^7,16,17^. These results implicate that the polarization of dγδ T cells may play an important role in regulation of immune response at the maternal-fetal interface. However, the related mechanism remains unclear.

Receptor activator for nuclear factor-κB (RANK) and its only known ligand tumor necrosis factor ligand superfamily member 11 (TNFSF11, also known as RANKL) have dual roles in immune regulation. On the one hand, they promote adaptive immune response by inducing the production of IL-12 in mature dendritic cells and polarization of CD4^+^ T cells into Th1 cells^18^. On the other hand, they exert their immunosuppression through inducing the polarization of regulatory T cells and participating in the establishment of central as well as peripheral tolerance^19^. In our previous studies, RANKL/RANK has been identified and functionally described at the maternal-fetal interface where it involved in the maintenance of pregnancy by promoting the growth of decidual stromal cells (DSCs) and inducing decidual M2 macrophage polarization^20,21^. However, to date there have no studies about the effects of RANKL/RANK interaction on dγδ T cells.

In this article, we focus on the interaction between DSCs-derived RANKL and RANK expressed on dγδ T cells and reveal their role in the maintenance of early pregnancy and RSA.

## Results

### The abnormal low level of RANKL/RANK at the maternal-fetal interface in RSA patients

**Fig. 1:**
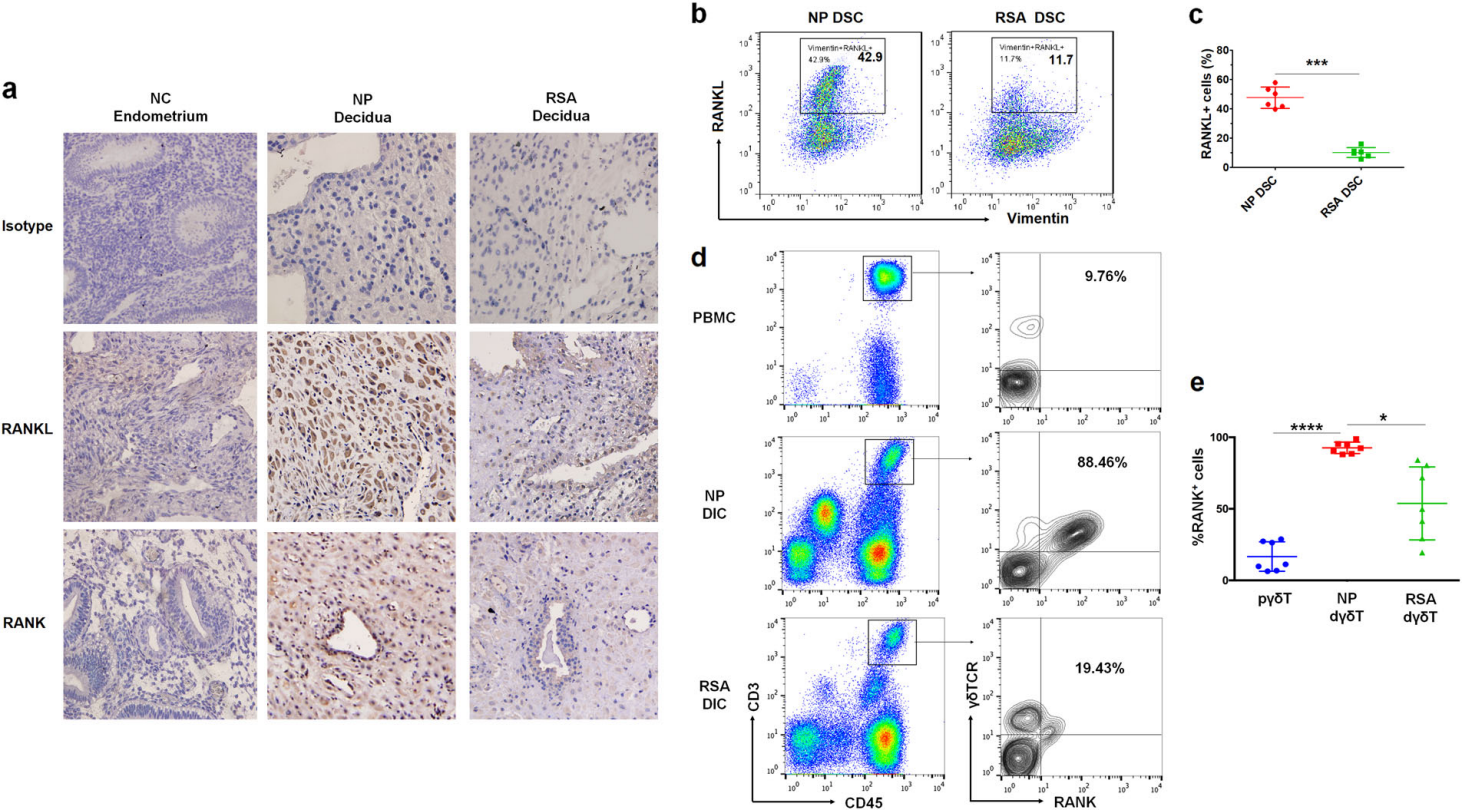
Expressions of RANK/RANKL at the maternal-fetal interface. **a** Immunohistochemistry analysis of RANKL and RANK expression in decidua from normal pregnancy (*n* = 10), control endometrium from non-pregnant women (NP, *n* = 10), and decidua from women with recurrent spontaneous abortion (RSA) (*n* = 8). Original magnification: ×200. **b**, **c** Flow cytometry (FCM) analysis of membrane RANKL expression on decidual stromal cells (DSCs) from women with NP decidua (*n* = 6) and RSA decidua (*n* = 6). **d**, **e** FCM analysis of RANK expression on γδ T cells in peripheral blood (*n* = 7), NP decidua (*n* = 7), and RSA decidua (n = 7). Data are shown as the mean ± SD. *P < 0.05, ***P < 0.001, and ****P < 0.0001 (Student’s *t-*test or Kruskal–Wallis H test). *NC* normal control, *PBMC* peripheral blood mononuclear cells, *DIC* decidual immune cell, *pγδ T* γδ T cells from peripheral blood, *dγδ T* γδ T cells from decidua

To investigate the interaction between DSC-derived RANKL and RANK expressed on dγδ T, we first analyzed the expression of RANKL and RANK in decidua during early pregnancy. As shown, the strong positive staining of RANKL and RANK located in the cytoplasm and cell membrane of DSCs was observed by immunohistochemistry (Fig. 1a). RANKL and RANK expression in decidua from normal pregnancy were significantly higher than that in control endometrium from non-pregnant women (Fig. 1a). Further analysis showed that DSCs from normal pregnancy had a higher level of membrane RANK (Fig. 1b, c). Flow cytometry analysis revealed high levels of RANK expression on dγδ T cells, as the percentage of RANK^+^ γδ T cells (CD45^+^CD3^+^γδTCR^+^) was over 90% at the maternal-fetal interface, while less than 10% of peripheral blood (Fig. 1d, e). The tissue-specific high expression level of RANK on dγδ T suggests the possible role of RANK in the regulation of dγδ T and maternal-fetal immunotolerance.

Meanwhile, we investigated the relationship between RANKL/RANK signaling in decidua and the occurrence of RSA. Interestingly, by comparison with normal pregnant women, RSA patients showed weaker levels in decidua for the RANKL and RANK staining (Fig. 1a) and membrane RANKL expression (Fig. 1b, c). In accordance with this, a significant decrease in the percentage of RANK^+^ γδ T cells was observed in patients with RSA (53.79% ± 9.647) in first trimester compared with normal pregnancy (92.65% ± 1.517) (Fig. 1d, e). These findings indicate that the suppression of RANKL/RANK signaling at the maternal-fetal interface may be related to RSA.

### DSCs upregulate the expression of RANK on dγδ T cells

**Fig. 2:**
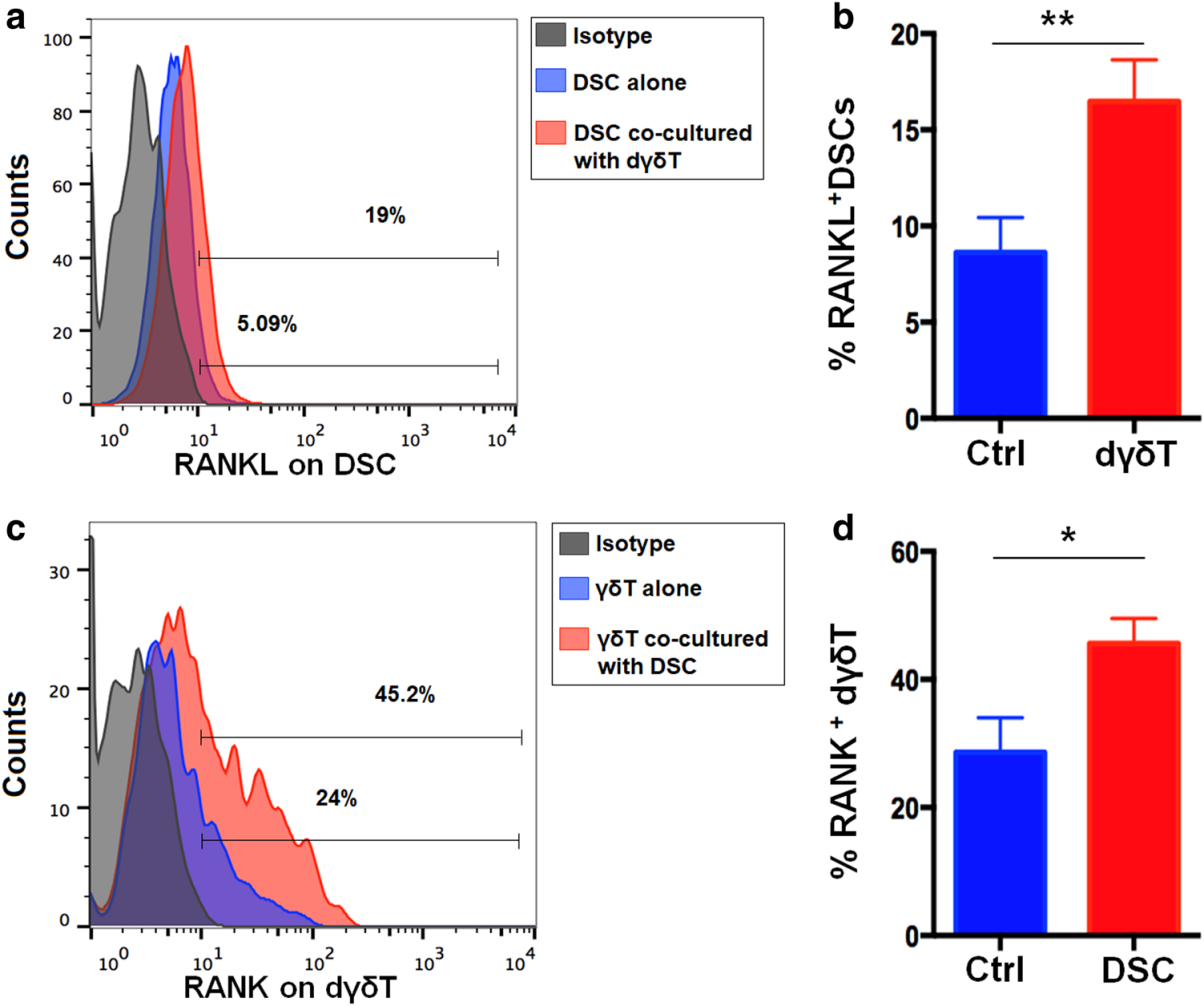
The interaction of DSCs and dγδ T cells promotes RANKL/RANK expression at the maternal-fetal interface. **a**, **b** RANKL expression on DSCs by FCM analysis after culture with or without the presence of dγδ T cells (*n* = 6) for 48 h. **c**, **d** RANK expression on dγδ T cells by FCM analysis after culture with or without the presence of DSCs (*n* = 6) for 48 h. Data are shown as the mean ± SD. *P < 0.05 and **P < 0.01 (Paired samples *t*-tests or Wilcoxon matched-pair signed-rank test)

To investigate the potential relationship between high level of RANKL/RANK, and the interaction of DSCs and dγδ T cells at the maternal-fetal interface, we constructed the co-culture model with DSCs and dγδ T cells for imitating local microenvironment of decidua during early pregnancy. FCM analysis revealed markedly increased expression of RANKL in DSCs (Fig. 2a, b) and RANK on dγδ T cells (Fig. 2c, d) in the co-culture unit. After culture alone for 48 h, the percentage of RANKL^+^ DSCs were reduced to 8.62% and increased to about two times with the presence of dγδ T cells (Fig. 2a, b). Meanwhile, the presence of DSCs changed the frequency of RANK^+^ dγδ T cells from 28.60 to 45.63% after culture for 48 h (Fig. 2c, d). Taken together, the interaction of DSCs and dγδ T cells maintains the high levels of RANKL/RANK at the maternal-fetal interface.

### RANK^+^ dγδ T cells present the activated phenotype

**Fig. 3:**
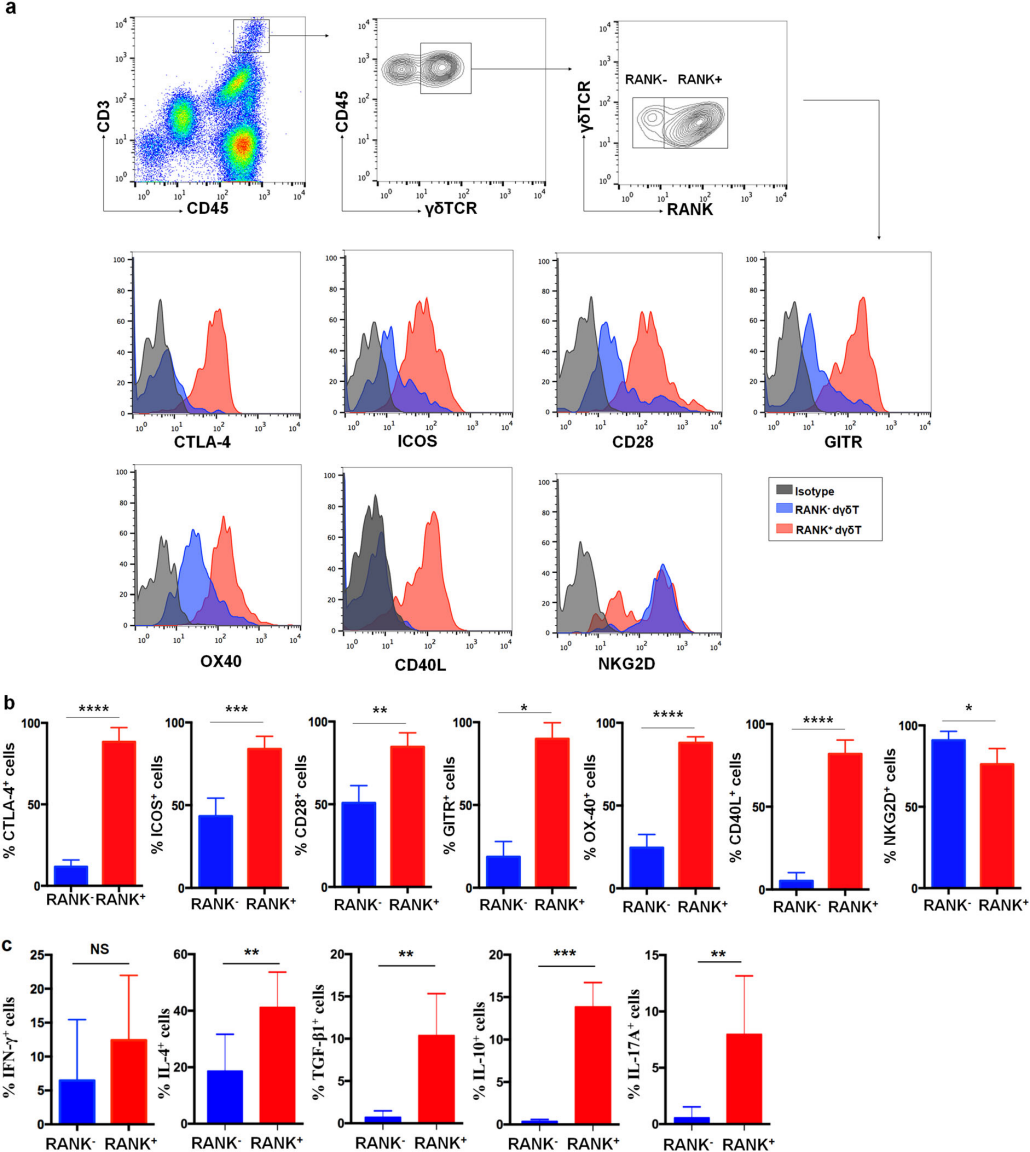
Phenotypic analysis and cytokine profile of decidual RANK^+^ and RANK^−^ γδ T cells. **a–c** The expression levels of costimulatory molecules (e.g., CTLA-4, OX-40, GITR, CD28, ICOS, and CD40), a cytotoxicity marker NKG2D, and cytokines (e.g., IFN-γ, IL-4, TGF-β1, IL-10, and IL-17A) on decidual RANK^−^ dγδ T cells and RANK^+^ dγδ T cells (*n* = 6) by FCM analysis. Data are shown as the mean ± SD. *P *<* 0.05, **P < 0.01, ***P < 0.001, and ****P < 0.0001; *NS* no statistically difference (Paired samples *t*-tests or Wilcoxon matched-pair signed-rank test)

Our further phenotypic analysis showed that both RANK^+^ pγδ T and RANK^+^ dγδ T cells expressed higher levels of a series of costimulatory molecules, including CTLA-4, OX-40, GITR, ICOS, and CD40, compared with RANK^−^ cells (Fig. 3a, b, Figure S1). On the contrary, the expression level of NKG2D, one of the cytotoxicity markers of γδ T^22^, was decreased in RANK^+^ cells as compared with RANK^−^ cells (Fig. 3a, b, Figure S1). Moreover, cytokine pattern was different between RANK^+^ γδ T cells and RANK^−^ γδ T cells. RANK^+^ pγδ T as well as RANK^+^ dγδ T produce higher levels of IL-4, TGF-β1, and IL-17A compared with RANK^−^ cells (Fig. 3a–c, Figure S1). In decidua, RANK^+^ γδ T produce higher level of IL-10 compared with RANK^−^ γδ T cells (Fig. 3a–c). However, such difference was not found in peripheral blood. Meanwhile, no significant difference in the production of IFN-γ between RANK^+^ and RANK^−^γδ T was detected (Figure S1). Taken together, RANK^+^ γδ T are more functionally activated than RANK^−^ γδ T.

### RANKL expressed on DSCs induce polarization of Foxp3^+^ regulatory γδ T cells and TGF-β1 production

**Fig. 4:**
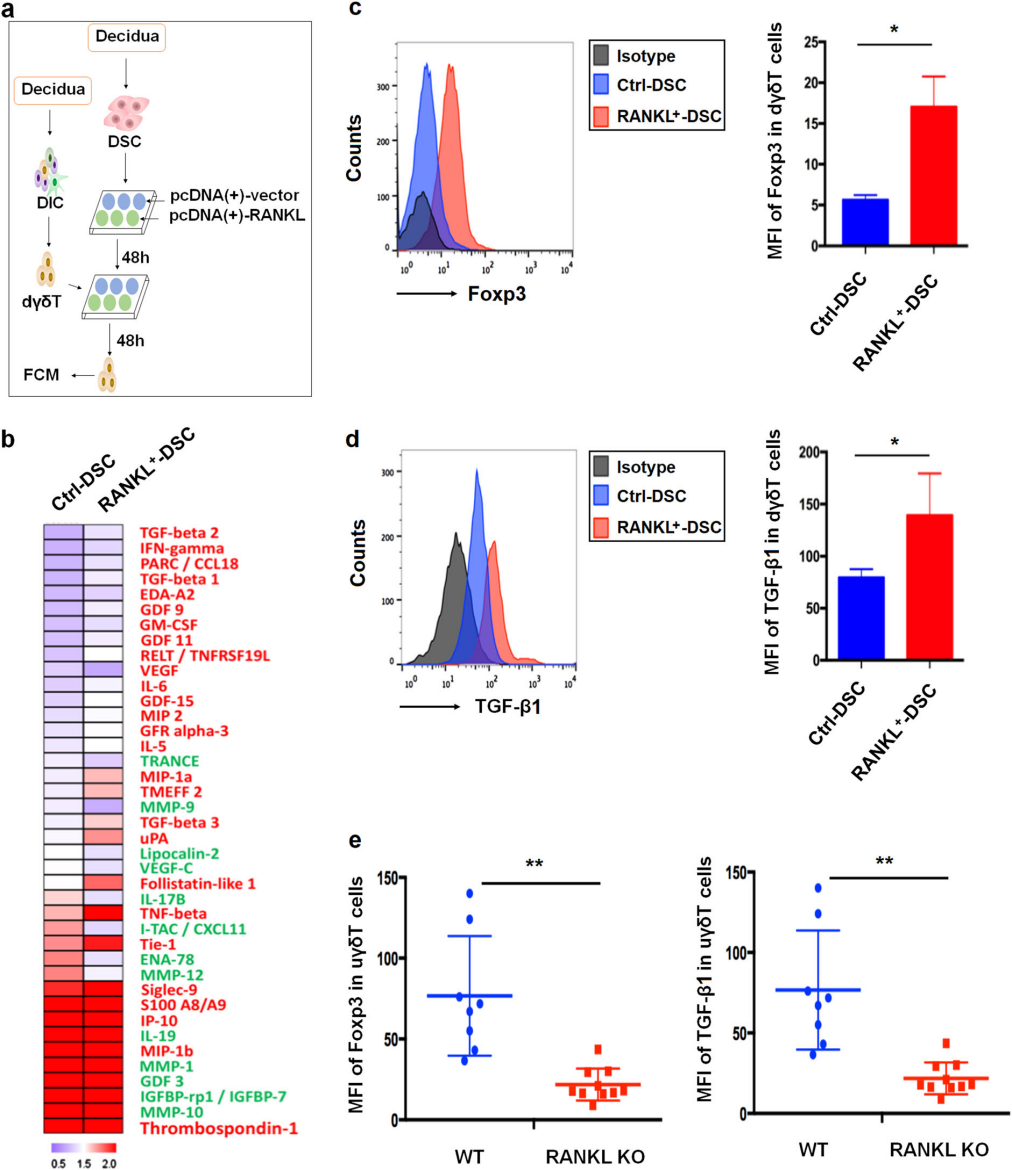
RANKL from DSCs induce polarization of Foxp3 + regulatory γδT cells and TGF-β1 production. **a** After co-culture with RANKL-overexpressed (RANKL^+^) or control (ctrl) DSCs at a 1:1 ratio for 48 h, the supernatants and decidual γδ T (dγδ T) cells were collected for protein array and flow cytometry analysis. **b** Heatmap of selected proteins differentially expressed in culture supernatants. **c**, **d** The median fluorescence intensity (MFI) of Foxp3 (**c**) and TGF-β1 (**d**) in dγδ T cells. **e** FCM analysis of MFI of Foxp3 and TGF-β1 in uterine γδ T cells from WT (*n* = 8) pregnant and RANKL^−/−^ (*n* = 10). *WT* wild-type, *KO* knockout. Data are shown as the mean ± SD. *P *<* 0.05 and **P < 0.01 (Wilcoxon matched-pair signed-ranks test or Student’s *t*-test with correction)

Different γδ T subsets are characterized by the secretion of cytokines and expression of transcription factors^9^. By analyzing their profiles of cytokines and transcription factors, we explored whether RANKL/RANK signaling involved in the polarization of dγδ T cells both in vitro and in vivo. To gain more insights into how RANKL overexpression affects the cytokine production of dγδ T cells, we directly co-cultured the γδTCR^+^ cells isolated from human decidua tissues with RANKL-overexpressed or control DSCs, and collected supernatant from co-culture system and subjected them to cytokines microarray analysis^23^ (Fig. 4a). In the co-culture system with RANKL overexpression, cytokines were more highly produced including several TGF-β superfamily members TGF-β1, 2, 3, as well as growth/differentiation factor (GDF) 9, 11, 15, and angiogenic factors vascular endothelial growth factor (VEGF) (Fig. 4b). Subsequently, FCM analysis showed that the secretion of cytokine TGF-β1 and expression of transcription factor Foxp3 were significantly increased in dγδ T cells after treated by RANKL-overexpressed DSCs as compared with control group (Fig. 4c, d). By contrast, there were no significant differences in other cytokines, including IFN-γ, TNF-α, IL-4, IL-17, IL-22, and IL-10, or transcription factors which include T-bet, GATA-3, RORC, AHR, and Bcl-6 (data not shown). Meanwhile, similar results were obtained when pγδ T cells co-cultured with RANKL-overexpressed or control DSCs (Figure S2).

By using RANKL knockout mice, we further verified the effect of RANKL/RANK interaction on γδ T cells at the maternal-fetal interface in vivo. Uterine γδ T cells (CD45^+^CD3^+^γδTCR^+^) from pregnant RANKL^−/−^ mice exhibited decreased Foxp3 expression and produced decreased amounts of TGF-β1 compared to cells from pregnant wild-type (WT) mice (Fig. 4e). Thus, decidual RANKL/RANK interaction has little impacts on the polarization of γδT cells toward other subgroups, except for TGF-β1-producing regulatory γδ T cells.

### Polarization of regulatory γδ T cells and TGF-β1 production induced by RANKL are dependent on the NF-κB pathway

**Fig. 5:**
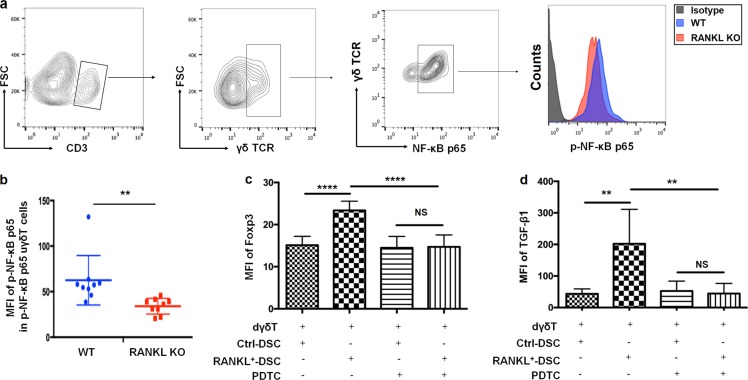
RANKL regulates TGF-β1-producing regulatory decidual γδT cells via the NF-κB pathway. **a**, **b** FCM analysis of the MFI of Phospho-NF-κB p65 in the uterine NF-κB p65^+^ γδ T cells form pregnant WT and RANKL^−/−^ mice (*n* = 9). **c**, **d** After 1-h pretreatment with or without pyrrolidine dithiocarbamate (PDTC, 100 μM) and 2-day co-culture with RANKL^+^ DSCs or ctrl DSCs, the MFI level of Foxp3 and TGF-β1 in d γδT (*n* = 8) were analyzed by FCM. Data are shown as the mean ± SD. **P < 0.01, ****P < 0.0001, and *NS* no significance (Wilcoxon rank-sum test or One-way ANOVA or Wilcoxon matched-pair signed-ranks test)

Recently, several studies discovered that nuclear factor kappa-light-chain-enhancer of activated B cells (NF-κB), the major downstream pathway of RANKL/RANK interaction, is critical to the expression of Foxp3, as well as the development and immune suppression of regulatory T cells^24,25^. As is known, when activated, NF-κB is phosphorylated for further nuclear entry^26^. To test whether RANKL/RANK signaling exerts effects on γδ T cells by activating NF-κB pathway, we assessed the phosphorylation status of NF-κB in γδ T cells. To this end, expression level of phospho-NF-κB p65 were analyzed after gating on NF-κB p65^+^ γδ T cells. As shown in Fig. 5a, b, at day 8.5 of gestation, uterine NF-κB p65^+^ γδ T cells expressed less phospho-NF-κB p65 in RANKL^−/−^ mice compared to WT controls. Thus, RANKL-deficiency reduces NF-κB activation in uterine γδ T cells.

We further proved that the activation of NF-κB is crucial for RANKL/RANK signaling to regulate dγδ T polarization with the help of PDTC, a selective inhibitor of NF-κB. As shown in Fig. 5c, d, increased levels of TGF-β1 production and Foxp3 expression in dγδ T, which was co-cultured with RANKL-overexpressed DSCs for 48 h, were almost completely aborted by PDTC. The pretreatment of human dγδ T with PDTC for 1 h before the co-culture, was capable of aborting the effects of RANKL/RANK interaction on dγδ T. These data suggest that NF-κB signaling pathway is involved in the RANKL-induced polarization of Foxp3 + dγδ T cells and TGF-β1 production.

### RANKL upregulates the expression of ICAM-1/VCAM-1 on DSCs and integrins on dγδ T cells

**Fig. 6:**
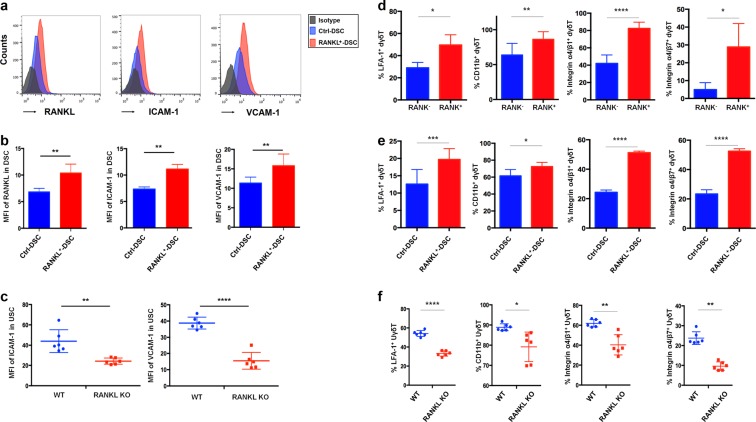
RANKL upregulates cell-adhesion molecule expression on DSCs and integrin expression on γδ T cells. **a**, **b** FCM analysis of ICAM-1 and VCAM-1 expression in RANKL^+^ DSCs or ctrl DSCs (*n* = 13) by FCM. **c** FCM analysis of ICAM-1 and VCAM-1 expression in uterine stromal cells of WT and RANKL^-/-^ mice (*n* = 6). **d** FCM analysis of integrins (e.g., LFA-1, CD11b, integrin α4β1, and integrin α4β7) on human RANK^+^ and RANK^−^dγδ T (*n* = 6). **e** FCM analysis of integrins on human dγδ T cells cultured with ctrl DSCs or RANKL^+^ DSCs (*n* = 6) for 48 h. **f** FCM analysis of integrins on uterine γδ T cells of pregnant WT and RANKL^-/-^ mice (*n* = 6). Data are shown as the mean ± SD. *P *<* 0.05, **P < 0.01, ***P < 0.001, and ****P < 0.0001 (Wilcoxon rank-sum test, Wilcoxon matched-pair signed-ranks test or Student’s *t*-test with or without correction)

Since RANKL enhances leukocyte residence by inducing adhesion molecules, including intercellular adhesion molecule 1 (ICAM-1) and vascular cell-adhesion molecule 1 (VCAM-1), in endothelial cells^27^, we hypothesized that DSC adhesion molecule expression and γδ T cell residence may also be regulated by RANKL. Importantly, in skin-draining lymph nodes, RANKL also upregulates cell-adhesion molecules in stromal cells^28^. As shown, overexpression of RANKL significantly upregulated ICAM-1 and VCAM-1 in human DSCs (Fig. 6a, b).

In contrast, RANKL deficiency led to the decreased ICAM-1 and VCAM-1 expression in murine uterine stromal cells at day 8.5 of gestation (Fig. 6c). Thus, RANKL promotes the expression of cell-adhesion molecules in stromal cells at the maternal-fetal interface both in vitro and in vivo.

As is known, binding to integrin is a prerequisite of cell-adhesion molecule to mediate cell migration. ICAM-1 binds to integrins of lymphocyte function-associated antigen 1 (LFA-1, also known as CD11a/CD18), or macrophage-1 antigen (Mac-1, also known as CD11b/CD18), and VCAM-1 binds to integrins of very late antigen-4 (VLA-4, also known as integrin α4β1, CD49d/CD29), or integrin α4β7. As shown, all integrins of ICMA-1 and VCAM-1 were expressed in γδ T cells of human decidua during early pregnancy (Fig. 6d). Of note, RANK^+^ dγδ T expressed higher levels of integrins, including LFA-1, CD11b, integrin α4β1, and integrin α4β7, compared with RANK^−^cells (Fig. 6d). The results of co-culture system in vitro showed that human dγδ T cells cultured with RANKL-overexpressed DSCs exhibited the increased integrins expression compared to cells cultured with control DSCs (Fig. 6e). Furthermore, significant reductions in integrins expression were observed in uterine γδ T cells of pregnant RANKL-deficient mice (Fig. 6f). Thus, these results indicate that DSC-derived RANKL upregulates cell-adhesion molecule expression on DSCs, as well as integrin expression on γδ T cells, and may further enhance γδ T cell residence into decidua.

### The RANKL/RANK interaction enhances the residence of γδ T cell into decidua

**Fig. 7:**
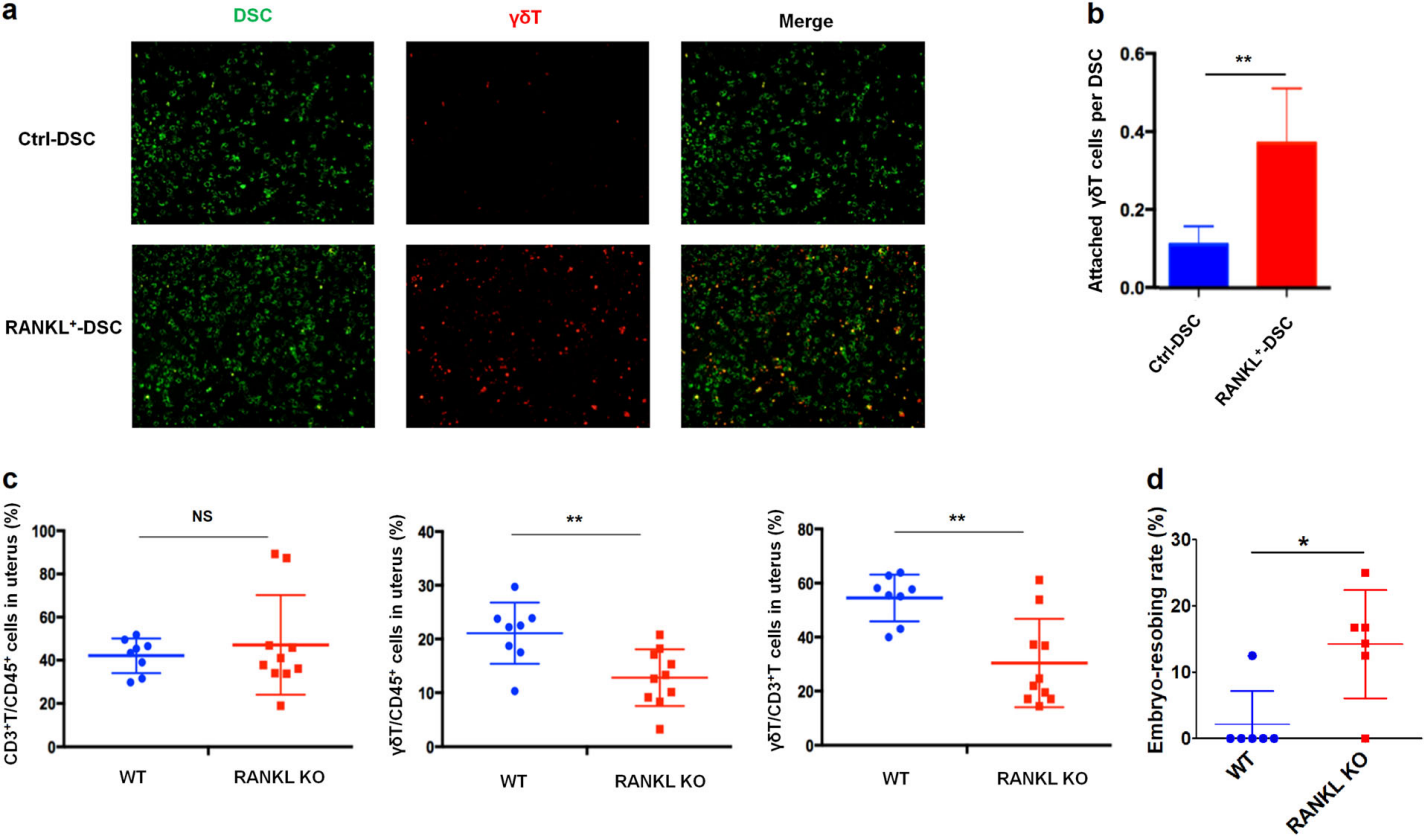
RANKL/RANK interaction enhances the residence of γδ T cell into decidua. **a** Representative fluorescence images for PKH-67 labelled DSCs (green), attached PKH-26 labeled dγδ T cells (red), and their overlaid images. **b** Quantitative assay of adhesion of decidual γδ T (dγδ T) cells on DSCs after co-culture with RANKL^+^ DSCs or ctrl DSCs (*n* = 6) for 24 h. **c** Percentages of γδ T cells (γδTCR^+^CD3^+^CD45^+^) and T cells (CD3^+^CD45^+^) from uteruses of pregnant WT (*n* = 8) and RANKL^−/−^ (*n* = 10) mice. **d** The embryo absorption rate in pregnant WT and RANKL^−/−^ mice (n = 6) was counted on day 14 of gestation. Data are shown as the mean ± SD. *P *<* 0.05, **P < 0.01 (Student’s *t*-test)

Considering ICAM-1 and VCAM-1 are important in leukocyte migration and residence, firstly we assessed the effect of RANKL on cell adhesion between DSCs and γδ T cells by the adhesion experiments in vitro. As shown, overexpression of RANKL significantly increased the ability of DSCs to attract PKH-26-labeled γδ T cells isolated from human decidua tissues (Fig. 7a, b). In support of these data, uterine immune cell populations in mice at day 8.5 of gestation were observed. Compared to WT mice, RANKL^−/−^ mice had reduced numbers and frequencies of uterine γδ T cells, while the total number of T cells remained unaltered (Fig. 7c). Meanwhile, normal frequencies of other immune cell populations, including natural killer cells, natural killer T cells and macrophages, were present in the uterine of RANKL^−/−^ mice during early pregnancy (data not shown). Of note, the lack of RANKL resulted in the increase of mouse embryo absorption (Fig. 7d). These findings indicate that the RANKL/RANK interaction increases the residence of γδ T cell into decidua in early pregnancy, and contributes to the establishment and maintenance of normal pregnancy.

## Discussion

Here we demonstrated that, at the maternal-fetal interface, DSC-derived RANKL promotes the polarization of dγδ T cells into Foxp3^+^ regulatory γδ T cells and elevates TGF-β1 production by activating NF-κB pathway. Moreover, RANKL enhances the aggregation and residence of γδ T cells into decidua by upregulating the expression of cell-adhesion molecules. Taken together, RANKL/RANK interaction contributes to the maternal-fetal tolerance by strengthening the crosstalk between DSCs and dγδ T cells.

RANKL and RANK have been recently identified as key determinants of osteoclastogenesis. Hormones, cytokines, and other humoral factors influence osteoclastogenesis within the tumor/bone microenvironment primarily through increases in RANKL^29^. In breast cancer, tumor-derived factors, including parathyroid hormone-releasing protein (PTHrP), prostaglandin E_2_ (PGE_2_), TNFα, and interleukins (e.g, IL-1, IL-6, IL-11), have been demonstrated to enhance RANKL expression by osteoblasts and other bone stromal cells present, which may directly contribute to osteoclastogenesis^29,30^. In this study, the co-culture of DSCs and γδ T cells led to the high levels of RANKL on DSCs and RANK on γδ T cells, suggesting the cytokines secreted by DSCs and γδ T cells may contribute to this regulatory process. Owing to the important role of PGE_2_, IL-1β, and IL-11 in the decidualization^31–35^, it can be speculated that local microenvironment at maternal-fetal interface, including hormones, DSC, and DIC-derived cytokines, is bound to play an important role in maintaining high levels of RANKL; however, the specific mechanism remains to be studied.

In comparison with RANK^−^ dγδ T cells, RANK^+^ cells tend to be more active based on their higher expression level of costimulatory molecules and increased secretion of cytokines. Remarkably, among all seven tested costimulatory molecules, only NKG2D decreased in RANK^+^ dγδ T cells compared to that in RANK^−^ cells. As the most widely studied NK receptor (NKR) in γδ T cells, NKG2D has been proved to mediate cytotoxicity of γδ T cells and tissue damage^36,37^. It is possible that, during early pregnancy, these highly activated RANK^+^ dγδ T cells play a role mainly in cytokine production instead of cell-killing at the maternal-fetal interface. Moreover, the increased expression level of CTLA-4 in RANK^+^ γδ T cells is crucial in immunosuppression mediated by T cell^38^.

We found that, during early pregnancy, RANKL plays a two-pronged strategy, which involves inducing polarization of Foxp3^+^ regulatory γδ T cells through the highly expressed receptor RANK on γδ T cells (over 90% are RANK^+^) and enhancing γδ T cell residence into decidua. However, stimulation with recombinant human RANKL protein did not change the differentiation of regulatory γδ T cells (data not shown), suggesting that the effect of DSCs on regulatory γδ T cells polarization mainly depends on membrane RANKL. Previously, Foxp3^+^ T cells have been demonstrated to play a role in inducing early maternal tolerance^39^. In addition, the RANKL/RANK interaction promoted TGF-β1 production of dγδ T cells. These processes are possibly dependent on the NF-κB signaling pathway. TGF-β can directly inhibit the cytotoxicity and activity of effector cells at the maternal-fetal interface, including promotes the conversion of CD16^+^ NK cells into CD16^−^ NK cells^40^, downregulates the expression level of CD80/CD86 on macrophages^41^, and limits IL-17 production in Th17 cells as well as T-bet expression and IFN-γ production in Th1 cells^42^. At the same time, TGF-β can in turn induces the polarization of Foxp3^+^ regulatory T cells^43^, as well as protect them from apoptosis^44^, and thus forms a positive feedback to suppress anti-fetal responses. Moreover, uterine γδ T cells have been shown to prevent fetal rejection through TGF-β production at the maternal-fetal interface^45^, which also consists with our results. In our previous study, we found that the depletion of macrophage in pregnant mice led to a significant decrease in RANKL on uterine DSCs, and adoptive transfer of RANK^+^ macrophages relieves murine embryo absorption induced by macrophage depletion^21^. These results suggest that the fetal loss in RANKL^−/−^ mice is closely related to the abnormal polarization of decidual macrophages. Further, in this study, we found that, except for participating in the polarization of macrophages, RANKL also promotes the residence and polarization of TGF-β1-producing regulatory γδ T cells at the maternal-fetal interface. To sum up, RANKL/RANK interaction regulates the polarization, proliferation, immunoregulatory function, and residence of dγδ T cells, and may contribute to the suppression of fetal rejection as well as the maintenance of pregnancy during the first trimester, which needs further study.

At the maternal-fetal interface, the accumulation of γδ T cells is quite obvious during early pregnancy. In peripheral blood, γδ T cells are a minor subset of T cells (5–10%), while turns the major subset in decidua (over 60%)^6^. It has been reported that RANKL increases migration of human lung cancer cells through NF-κB-upregulated ICAM-1^46^. Our finding indicates that RANKL may also participate in γδ T cells accumulation regulation by enhancing the adhesion of γδ T cells to DSCs, and this effect should be dependent on the interaction of ICAM-1/VCAM-1 and integrins. However, the regulatory mechanism of RANKL on ICAM-1/VCAM-1 needs further research.

**Fig. 8:**
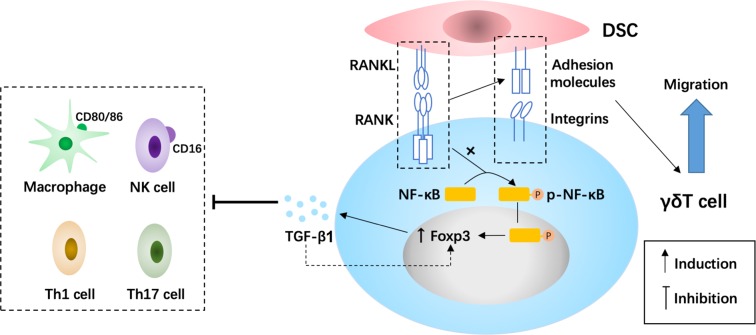
The schematic roles of RANKL in the regulation of TGF-β1-producing regulatory γδ T cell polarization at the maternal-fetal interface. At the maternal-fetal interface, DSC-derived RANKL enhances the accumulation of dγδ T cells by upregulating cell-adhesion molecule expression on DSCs and γδ T cells, and promotes the polarization of dγδ T cells into Foxp3^+^ regulatory cells possibly by activating NF-κB pathway and inducing TGF-β1 production in early pregnancy. Also, TGF-β1 may directly inhibit the cytotoxicity and activity of effector cells, including promotes the conversion of CD16^+^ NK cells into CD16^−^ NK cells, downregulates the expression level of CD80/CD86 on macrophages, and limits IL-17 production in helper T (Th)17 cells as well as T-bet expression and IFN-γ production in Th1 cells, which further contributes to maternal-fetal immune tolerance for the establishment and militainment of normal pregnancy

In summary, as shown in Fig. 8, our study demonstrated that during early pregnancy RANKL from DSCs, on the one hand, can induce the polarization of Foxp3^+^ regulatory γδT cells and TGF-β1 production possibly by NF-κB signaling pathway, on the other hand, can enhance the adhesion and residence of dγδ T cell into decidua. These integral effects will promote the resident and polarization of TGF-β1-producing regulatory γδT cells, and contribute to the maternal-fetal immune tolerance, and establishment and maintenance of pregnancy. In contrast, an abnormal low expression of RANKL and RANK at the maternal-fetal interface leads to the deficiency of regulatory γδT cells and TGF-β1 production and decidual macrophage dysfunction, and enhances the risk of RSA occurrence.

## Materials and methods

### Human samples and cell isolation

Decidua samples were obtained from 72 women with clinically normal pregnancy requiring pregnancy termination (age: 28.13 ± 4.37 years; gestational age at sampling: 50.92 ± 5.26 days; mean ± SD) and 21 women with recurrent spontaneous abortions (age: 29.12 ± 3.09 years; gestational age at sampling: 61.04 ± 8.12 days; mean ± SD). Abortions caused by genetic, endocrine, or anatomical abnormalities, as well as infection, were excluded. Normal endometrium was obtained at hysterectomy from patients with uterine leiomyoma (*n* = 10) as healthy controls. None of the included patients had experienced complications related to pelvic inflammatory disease and none took any medications or received hormonal therapy within 6 months before surgery. The Ethics Committee of Obstetrics and Gynecology Hospital, Fudan University approved the study, and tissues were obtained with informed consent from each patient before surgery.

Fresh decidua tissues were washed in phosphate buffer saline (PBS) and cut into pieces within 30 min after operation. DICs and DSCs were then isolated from the shredded tissue as previously described^47^. DICs and DSCs used in this study were isolated from the tissues of normal pregnancy recurrent spontaneous abortions.

Peripheral blood was collected from 26 healthy donors. Peripheral blood mononuclear cells (PBMCs) were isolated from peripheral blood by Ficoll-Paque (GE Healthcare) centrifugation.

### Mice

Male and female RANKL heterozygote mice were purchased from the Jackson Laboratories (Sacramento, CA, USA). RANKL^−/−^ mice and WT littermates were generated by mating RANKL heterozygote. All mice were kept in the Laboratory Animal Facility of Fudan University (Shanghai, China). Age-matched (8 weeks) pregnancy mice were used in this study. The day of appearance of a copulatory plug was arbitrarily designated as day 0 of gestation. Uteruses were collected from RANKL^−/−^ or WT littermates at day 8.5 of gestation. All procedures involving animals were approved by the Animal Care and Use Committee of Fudan University.

### Primary cell cultures

Primary human and mice immune cells were cultured in RPMI 1640 (Gibco, Grand Island, NY, USA) with 10% fetal bovine serum (FBS; Gibco, Grand Island, NY, USA), 100 IU/mL penicillin, 100 μg/mL streptomycin, 250 ng/mL amphotericin B, and 20 ng/mL recombinant human IL-2. Primary human and mice stromal cells were cultured in Dulbecco’s Modified Eagle Media: Nutrient Mixture F-12 (DMEM/F-12; Gibco, Grand Island, NY, USA) with 10% fetal bovine serum (FBS; Gibco, Grand Island, NY, USA), 100 IU/mL penicillin, 100 μg/mL streptomycin, and 250 ng/mL amphotericin B.

### Flow cytometry

For flow cytometry (FCM) analyses, cells were incubated with monoclonal antibodies (Table S1) according to the manufacturer’s instructions. Intracellular cytokines were detected after stimulating with Cell Activation Cocktail (Biolegend) for 6 h. Intracellular proteins or intranuclear transcription factors were stained using Fixation/Permeabilization Solution Kit (BD Pharmingen) or Transcription Factor Buffer Set (BD Pharmingen), respectively. All samples were run on a Beckman-Coulter CyAN ADP Analyzer (Beckman-Coulter, Brea, CA, USA), and data were analyzed by FlowJo software (TreeStar, Ashland, OR, USA).

### Immunohistochemistry

Anti-RANKL antibody (R&D Systems, Abingdon, UK) was used for human decidua and endometrium immunohistological staining according to a previously described method^47^.

### Purification of γδ T cells

Human γδ T cells were isolated from DICs or PBMCs using Anti-γδTCR MicroBead Kit (Miltenyi Biotec, Bergisch Gladbach, Germany) according to the manufacturer’s instructions, and a 95% purity of CD45^+^CD3^+^γδTCR cells was confirmed by flow cytometry.

### Co-culture of DSCs and γδ T cells

DSCs and dγδ T cells isolated from decidual of normal pregnancy were used to construct the co-culture system in vitro. More concretely, DSCs were cultured in a 6-well plate (5 × 10^5^ cells per well) for overnight, and then co-cultured with or without freshly isolated dγδ T cells at a 1:1 ratio. In the meantime, part of the isolated dγδ T cells were cultured in a 6-well plate (5 × 10^5^ cells per well) without DSCs. After 48 h, DSCs and dγδ T cells were collected for further flow cytometry analyses.

After 48 h of transfection with pcDNA(+)-RANKL or pcDNA(+)-vector plasmid (GeneChem Co., Ltd, Shanghai, China), DSCs were directly co-cultured with freshly isolated decidual/peripheral γδ T cells at a 1:1 ratio. In addition, in some groups, dγδ T cells were pretreated with or without 100 μM pyrrolidine dithiocarbamate (PDTC; MedChemExpress, USA) for 1 h, and co-cultured with RANKL-overexpressed or control DSCs. After 48 h of co-culture, γδ T cells were collected for further flow cytometry analyses.

### Protein array

After cultured in 5% FBS medium for an extra day, supernatants were collected from the DSC (from three women with normal pregnancy, mixed in the ratio of 1:1:1) -dγδ T co-culture system. The expression levels of over 500 proteins (including cytokines, chemokines, growth factors, angiogenic factors, etc.) were tested using L-Series Antibody Array 507 (RayBiotech) by the H-Wayen Biotechnologies in Shanghai.

### Adhesion experiment

After 2 days of transfection, RANKL-overexpressed or control DSCs were collected, washed, then stained with PKH-67 (Sigma), and cultured in a 96-well plate (3 × 10^4^ cells per well) for overnight. After that, PKH-26- (Sigma) labeled dγδ T cells were co-cultured with the treaded DSCs for 24 h. Then, to remove unattached dγδ T cells, the media was removed, and cells were washed gently with PBS twice. The results were obtained under a fluorescent microscope (Olympus, Tokyo, Japan) at five random fields of view and showed as the ratio of dγδ T cells to DSCs.

### Statistical analysis

The continuous variable is shown as the mean ± S.D. Variables between two dependent groups were analyzed by paired or unpaired samples *t*-test when the data are normally distributed and by Wilcoxon matched-pair signed-ranks test when the data are not normally distributed. As for independent samples, Student’s *t*-test with or without correction, Wilcoxon rank-sum test, one-way ANOVA, or Kruskal–Wallis H test was used according to the analysis of normal distribution and variance homogeneity. All analyses were conducted with Graphpad Prism software (La Jolla, CA, USA). *P* < 0.05 (*) was considered statistically significant.

## Supplementary information

Supplementary Table 1

Supplementary information

Supplementary Figure 1

Supplementary Figure 2
