# Supplementary material for: Decidual RANKL/RANK interaction promotes the residence and polarization of TGF-β1-producing regulatory γδ T cells
Source: Cell Death Dis. 2019 Feb 8;10(2):113. doi: 10.1038/s41419-019-1380-0 (PMC6368618; doi:10.1038/s41419-019-1380-0)
Supplement: Supplementary file 1 — Supplementary Table 1 [file 41419_2019_1380_MOESM1_ESM.docx]

| Antibodies | Source | Identifier |
| --- | --- | --- |
| APC anti-human CD3 | Biolegend | Cat#300312 |
| FITC anti-human TCR γ/δ | Biolegend | Cat#331208 |
| eFluor 450 anti-human CD45 | eBioscience | Cat#48-0459-41 |
| PE anti-human RANK/TNFRSF11A | R&D Systems | Cat#FAB683P |
| eFluor 450 anti-human CD3 | eBioscience | Cat#48-0037-42 |
| APC anti-human CD28 | Biolegend | Cat#302911 |
| APC anti-human CD314 (NKG2D) | Biolegend | Cat#320808 |
| PE/Cy7 anti-human/mouse/rat CD278 (ICOS) | Biolegend | Cat#313519 |
| APC anti-human CD134 (OX40) | Biolegend | Cat#350007 |
| PE/Cy7 anti-human CD154 | Biolegend | Cat#310831 |
| PE/Cy7 anti-human CD152 (CTLA-4) | Biolegend | Cat#349913 |
| PE/Cy7 anti-human CD357 (GITR) | Biolegend | Cat#371223 |
| APC anti-human IL-10 | Biolegend | Cat#501409 |
| APC anti-human IL-17A | Biolegend | Cat#512334 |
| APC anti-human IL-13 | Biolegend | Cat#501907 |
| APC anti-human LAP (TGF-β1) | Biolegend | Cat#349608 |
| PE/Cy7 anti-human IL-10 | Biolegend | Cat#501420 |
| PE/Cy7 anti-human IL-4 | Biolegend | Cat#500824 |
| APC anti-human IFN-γ | Biolegend | Cat#502511 |
| PE anti-human TNF-α | Biolegend | Cat#502909 |
| PE/Cy7 anti-human/mouse T-bet | Biolegend | Cat#644823 |
| PE anti-human/mouse ROR gamma t | R&D Systems | Cat#IC6006P-100 |
| PE/Cy7 anti-human/mouse Bcl-6 | Biolegend | Cat#358512 |
| APC anti-human/mouse GATA3 | Biolegend | Cat#653806 |
| Alexa Fluor 647 anti-mouse/rat/human FOXP3 | Biolegend | Cat#320014 |
| PE anti-human AHR | eBioscience | Cat#12-9854-42 |
| Pacific Blue anti-human CD54 | Biolegend | Cat#353109 |
| PE/Cy7 anti-human CD106 | eBioscience | Cat#25-1069-41 |
| [PE anti-mouse NF-κB p65](http://www.univ-bio.com/goods.php?id=356774) | CST | Cat#9460S |
| Alexa Fluor 647 anti-mouse Phospho-NF-κB p65 | CST | Cat#4887S |
| APC anti-human CD254 (TRANCE, RANKL) | Biolegend | Cat#347507 |
| APC anti-human/mouse/rat Vimentin | R&D Systems | Cat#IC2105A |
| PE anti-mouse CD106 | BD Pharmingen | Cat#561613 |
| Brilliant Violet 421 anti-mouse CD54 | BD Pharmingen | Cat#564704 |
| Alexa Fluore 750 anti-human/mouse/rat Foxp3 | R&D Systems | Cat#IC8970S |
| PE anti-mouse CD4 | Biolegend | Cat#100407 |
| APC anti-mouse IFN-γ | Biolegend | Cat#505809 |
| PE/Cy7 anti-mouse IL-4 | Biolegend | Cat#504117 |
| APC anti-mouse LAP (TGF-β1) | Biolegend | Cat#141405 |
| Brilliant Violet 421 anti-mouse CD3 | Biolegend | Cat#100227 |
| PE/Cy7 anti-mouse IL-10 | Biolegend | Cat#505025 |
| FITC anti-mouse TCRγ/δ | eBioscience | Cat#11-5811-81 |
| FITC anti-mouse CD8a | Biolegend | Cat#100705 |
| PE/Cy7 anti-mouse NK-1.1 | Biolegend | Cat#108713 |
| APC anti-mouse F4/80 | Biolegend | Cat#123115 |
| Brilliant Violet 510 anti-mouse CD45 | Biolegend | Cat#103138 |
| APC anti-human CD11b | Biolegend | Cat#301309 |
| APC anti-human CD29 | Biolegend | Cat#303007 |
| Brilliant Violet 421 anti-human Integrin beta 7 | BD Pharmingen | Cat#564283 |
| PE anti-human CD49d | Biolegend | Cat#304303 |
| PE/Cy7 anti-human CD18 | Biolegend | Cat#302117 |
| APC anti-mouse/human CD11b | Biolegend | Cat#101211 |
| APC anti-mouse/rat CD29 | Biolegend | Cat#102215 |
| Alexa Fluor 647 anti-mouse CD18 | Biolegend | Cat#101414 |
| PE anti-mouse CD49d | Biolegend | Cat#103607 |
| PE/Cy7 anti-human CD11a/CD18(LFA-1) | Biolegend | Cat#363418 |
| PE/Cy7 anti-mouse CD11a/CD18 (LFA-1) | Biolegend | Cat#141011 |
| FITC anti-mouse CD4 | Biolegend | Cat#100509 |
| Alexa Fluor 700 anti-mouse Integrin beta 7 | R&D Systems | Cat#FAB3060N |
| Brilliant Violet 510 anti-human CD49d | Biolegend | Cat#304317 |
