# Supplementary material for: Decidual RANKL/RANK interaction promotes the residence and polarization of TGF-β1-producing regulatory γδ T cells
Source: Cell Death Dis. 2019 Feb 8;10(2):113. doi: 10.1038/s41419-019-1380-0 (PMC6368618; doi:10.1038/s41419-019-1380-0)
Supplement: Supplementary file 2 — Supplementary information [file 41419_2019_1380_MOESM2_ESM.docx]

**Figure legends**

**sFigure 1** **Phenotypic analysis and cytokine profile of peripheral RANK^+^ and RANK^−^ γδ T cells**. (**a-c**) The expression levels of costimulatory molecules (CTLA-4, OX-40, GITR, CD28, ICOS and CD40), a cytotoxicity marker NKG2D, and cytokines (IFN-γ, IL-4, TGF-β, IL-10 and IL-17A) in RANK^-^ γδ T cells and RANK^+^ dγδ T cells from peripheral blood (n = 4 or 6) by FCM analysis. Data are shown as the mean ± SD. **P<*0.05, ***P*<0.01, ****P*<0.001 and *****P*<0.0001; NS, no statistically difference (*Paired samples t-test or Wilcoxon matched-pair signed-ranks test*).

**sFigure 2 RANKL from DSCs induce differentiation of Foxp3^+^ regulatory γδT cells and TGF-β production from peripheral blood**. After co-culture with RANKL-overexpressed (RANKL^+^) or control (ctrl) DSCs at a 1:1 ratio for 48 hours, the median fluorescence intensity (MFI) of Foxp3 and TGF-β in γδ T cells from peripheral blood (n = 5) were analyzed for FCM. pγδT: γδ T cells from peripheral blood. Data are shown as the mean ± SD. **P<*0.05 (*Paired samples* *t*-test).
