# Supplementary figures and images for: Decidual RANKL/RANK interaction promotes the residence and polarization of TGF-β1-producing regulatory γδ T cells
Source: Cell Death Dis. 2019 Feb 8;10(2):113. doi: 10.1038/s41419-019-1380-0 (PMC6368618; doi:10.1038/s41419-019-1380-0)

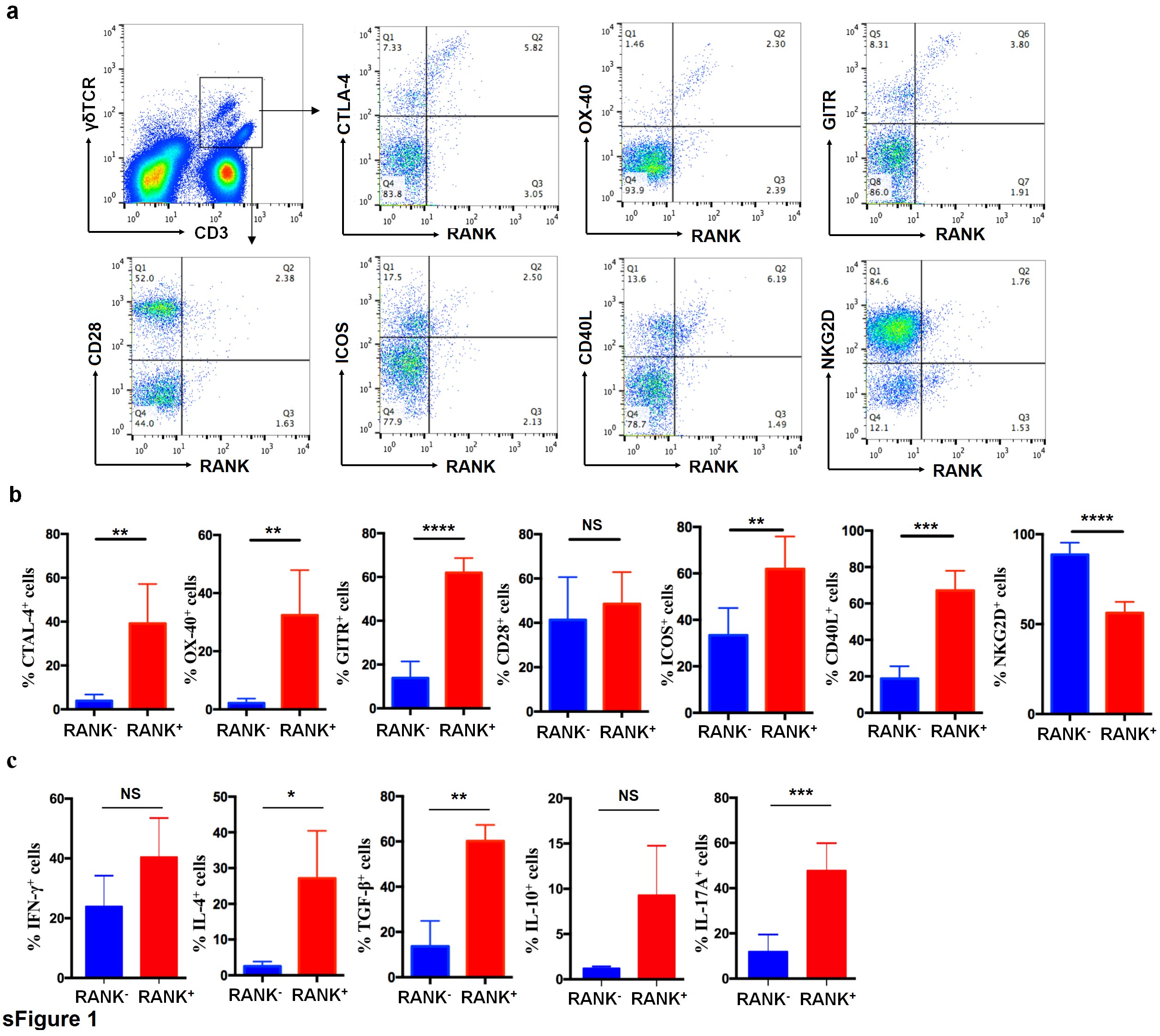

Supplement: Supplementary file 3 — Supplementary Figure 1 [file 41419_2019_1380_MOESM3_ESM.tif]

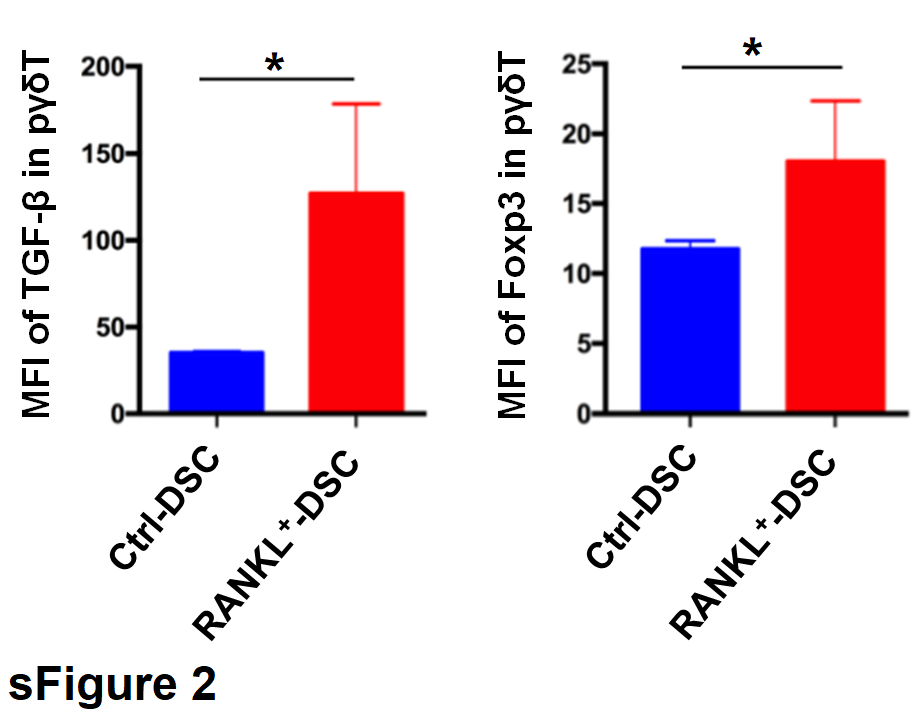

Supplement: Supplementary file 4 — Supplementary Figure 2 [file 41419_2019_1380_MOESM4_ESM.tif]
